# Reproductive factors and subsequent pregnancy outcomes in patients with prior pregnancy loss

**DOI:** 10.1186/s12884-024-06422-1

**Published:** 2024-03-25

**Authors:** Xin Yang, Fangxiang Mu, Jian Zhang, Liwei Yuan, Wei Zhang, Yanting Yang, Fang Wang

**Affiliations:** Reproductive Medicine Center, Second Hospital of Lanzhou University, No.82, Cuiying Road, Chengguan District, Lanzhou, 730030 Gansu Province China

**Keywords:** Pregnancy loss, Reproductive status, Pregnancy outcome, Logistic regression

## Abstract

**Background:**

At present, individualized interventions can be given to patients with a clear etiology of pregnancy loss to improve the subsequent pregnancy outcomes, but the current reproductive status of the patient cannot be changed. The aim of this study was to investigate the association between female reproductive status and subsequence pregnancy outcome in patients with prior pregnancy loss (PL).

**Methods:**

A prospective, dynamic population cohort study was carried out at the Second Hospital of Lanzhou University. From September 2019 to February 2022, a total of 1955 women with at least one previous PL were enrolled. Maternal reproductive status and subsequent reproductive outcomes were recorded through an electronic medical record system and follow-up. Logistic regression was used to evaluate the association between reproductive status and the risk of subsequent reproductive outcomes.

**Results:**

Among all patients, the rates of subsequent infertility, early PL, late PL, and live birth were 20.82%, 24.33%, 1.69% and 50.77% respectively. In logistic regression, we found that age (OR 1.08, 95% CI 1.04–1.13) and previous cesarean delivery history (OR 2.46, 95% CI 1.27–4.76) were risk factors for subsequent infertility in patients with PL. Age (OR 1.06, 95% CI 1.03–1.10), age at first pregnancy (OR 1.06, 95% CI 1.03–1.10), BMI (OR 1.06, 95% CI 1.02–1.11), previous PL numbers (OR 1.18, 95% CI 1.04–1.57) and without pre-pregnancy intervention (OR 1.77, 95% CI 1.35–2.24) were risk factors for non-live birth. Age (OR 1.06, 95% CI 1.03–1.09), age at first pregnancy (OR 1.06, 95% CI 1.02–1.09), BMI (OR 1.07, 95% CI 1.02–1.11), previous PL numbers (OR 1.15, 95% CI 1.02–1.31) and without pre-pregnancy intervention (OR 2.16, 95% CI 1.65–2.84) were risk factors for PL.

**Conclusions:**

The reproductive status of people with PL is strongly correlated with the outcome of subsequent pregnancies. Active pre-pregnancy intervention can improve the subsequent pregnancy outcome.

**Trial registration:**

This study was registered in the Chinese Clinical Trial Registry with the registration number of ChiCTR2000039414 (27/10/2020).

**Supplementary Information:**

The online version contains supplementary material available at 10.1186/s12884-024-06422-1.

## Background

Pregnancy loss (PL) is defined as the spontaneous demise of a pregnancy before the fetus reaches viability, which is a significant negative life event and impacts 10–15% of clinically recognized pregnancies. Recurrent pregnancy loss (RPL) refers to two or more consecutive PL episodes with the same sexual partner, accounting for approximately 1–2% [1, 2]. There are many reasons for the occurrence of RPL, including genetic abnormalities (fetal genetic abnormalities and parental genetic abnormalities), reproductive tract anatomical abnormalities, immune diseases, endocrine diseases, antiphospholipid syndrome, thrombotic disorders, and infections, but about 40-50% of the etiologies remain unexplained, Molecular mechanisms have not been fully explored, and these are defined as unexplained recurrent pregnancy loss (URPL) [3, 4]. In addition, PL was defined as primary if there without a previous ongoing pregnancy (viable pregnancy) beyond 24 weeks gestation, otherwise it was defined as secondary [1]. PL is a serious adverse event in life that greatly affects the physical and mental health of women. Women who experience PL have increased rates of anxiety and depression and other psychological disorders. It is reported that in RPL, the incidence of anxiety and depression in women can be as high as 47.7% and 51.7%, respectively [5]. At the same time, anxiety, and depression symptoms in women in early pregnancy are also risk factors for RPL [6].

In addition to influencing the etiology of pregnancy loss, personal factors (age, first pregnancy age, BMI) and reproductive status (total pregnancy number, pregnancy loss number, pregnancy type, induced abortion, live birth, ectopic pregnancies, molar pregnancy and, etc.) of the patient greatly influence the reproductive outcome [7]. Studies have found that age, the number of previous pregnancy loss and BMI are important influencing factors in pregnancy loss. The relationship between age and reproductive outcomes is well established, age-adjusted odds ratios for pregnancy loss were found to increase after each pregnancy loss and to be as high as 63% among women who had experienced six or more miscarriages [8]. However, the relationship between BMI and pregnancy outcomes remains controversial. Zhang et al. found that BMI ≥ 24.0 was associated with an increased risk of RPL. However, Lo and colleagues demonstrated that maternal obesity (BMI ≥ 30 kg/m2) significantly increased the risk of the disease miscarriage in couples with URPL, while there was no increased risk in women with overweight. Maconochie et al. found underweight (BMI < 18.5) was significantly associated with sporadic first trimester miscarriage, However, Lo et al. found that no increased risk of subsequent PL in women who are underweight as compared to women with normal BMI [9, 10].

Some differences were also found between primary and secondary PL, with secondary PL and ≥ 4 prior PL strongly associated with HLA-DRB1*03, and secondary PL of a boy from a previous birth has a negative impact on the outcome of subsequent pregnancies [11, 12]. Notably, patients with secondary PL had higher levels of tumor necrosis factor-α (TNF-α) in peripheral blood than primary PL, while high plasma TNF-α levels are reported to increase the risk of miscarriage in women with RPL [13]. This may indicate a higher risk of miscarriage in patients with secondary PL. It is also controversial whether previous induced abortion have an effect on subsequent PL. Infante-Rivard et al. found that induced abortion was a risk factor for subsequent PL, while Chung et al. found no statistical difference between induced abortion and PL risk [14, 15].

At present, some studies have found that reproductive history does not compromise subsequent live birth and perinatal outcomes in patients undergoing first frozen embryo transfer in in-vitro fertilization [16]. Whereas, a registry-based cohort study revealed that obstetric complications (still birth, ectopic pregnancies, and pregnancy losses) had a negative effect on the chance of live birth in the next pregnancy, and the identical pregnancy outcomes immediately preceding the next pregnancy had a larger impact than the total number of any outcome [17]. However, no studies have comprehensively evaluated reproductive factors and pregnancy outcomes in patients with prior PL.

Currently, individualized interventions can be given to patients with a clear etiology of PL to improve the outcome of subsequent pregnancies, but the current reproductive status of the patient cannot be changed. Therefore, this study aims to explore the relationship between reproductive factors and pregnancy outcomes in patients with prior PL.

## Methods

### Study population

A prospective, dynamic population cohort study was carried out at a university-affiliated fertility center. The cohort began in September 2019 and enrolled 1955 patients through February 2022. Written informed consent was obtained at the time of recruitment. Inclusion criteria: patients who had experienced at least one PL (diagnosis of PL according to the ESHRE, which spontaneous abortions prior to 24 weeks of gestation including biochemical pregnancy, and early PL was defined as PL before 10 weeks of gestational age [1]) and aged 18–42 years. Exclusion criteria: Patients who did not undergo any clinical examination after presentation and patients with severe psychiatric disorders who were not able to voluntarily enroll for subsequent follow-up. Patients are carefully asked for their reproductive history and personal demographic information when they join. If a patient had experienced a pregnancy loss and was currently non-pregnant at the time of presentation, an individualized pre-pregnancy intervention was given based on the results of the clinical examination. Pre-pregnancy interventions include improvements in thyroid function, correction of prothrombotic status, treatment of immune system disorders such as antiphospholipid antibody syndrome, folic acid supplementation, and advice on maintaining a healthy lifestyle. If a patient had experienced a pregnancy loss and was already pregnant at the time of presentation, pre-pregnancy intervention was lacking. During pregnancy, patients receive individualized treatment based on clinical symptoms and laboratory test results, including progesterone supplementation, aspirin, low molecular weight heparin, hydroxychloroquine, etc.

### Data collection

The population data was obtained from the Reproductive Medicine Middle School at the Second Hospital of Lanzhou University. Demographic information included age (< 25, 25–29, 30–34, ≥ 35), age at first pregnancy, BMI (< 18.5, 18.5–23.9, 24.0-27.9, ≥ 28), education and ethnicity. Pregnancy status data included the patient’s total number of previous pregnancies, the history of induced abortion, live birth (delivery method), birth defects, ectopic pregnancy, hydatidiform mole, previous PL numbers and pregnancy loss type (primary or secondary). Age at menarche, menstrual cycle, dysmenorrhea status and history of pelvic surgery were also collected. Each patient was followed up every 6 months after the first visit to track the patient’s pregnancy status, most recently in August 2022. At follow-up, we collected the outcome of the next pregnancy, the gestational age, delivery method, gender, birth weight of the live birth and whether the newborn was admitted to a neonatology department. Whether the mother had gestational diabetes mellitus, gestational hypertension, intrauterine cholestasis during pregnancy, and premature rupture. In addition, there are some patients in the follow-up process, both spouses want to have children, have normal sexual life, more than a year without contraception, but still do not conceive, we defined it as infertility [18]. We obtained information through a medical records registry and telephone follow-up.

### Statistical analysis

Descriptive statistics were used to describe the proportion and mean ± standard deviation of the demographic characteristics. Independent sample t test was used to compare the differences between the two groups, and one-way analysis of variance (ANOVA) was used to compare the differences among the three groups. Categorical data were compared with the chi-square test or Fisher’s exact test. The *P* < 0.1 of the variables were included in the Logistic regression analysis to estimate the odds ratio (OR) between research factors and risk of pregnancy outcome.

## Results

### Characteristics of participants

From all participants, 1955 patients were enrolled into our database between September 2019 to February 2022. Table 1 shows that the average age is 30.51 ± 4.41 years and the average of first pregnancy age is 26.41 ± 3.74 years. The proportion of overweight [(BMI 24.0-27.9 kg/m^2^)/ obesity (BMI ≥ 28 kg/m^2^) was diagnosed according to the Working Group on Obesity in China [19])] was 26.13%. Only one PL accounted for 40.87% and the RPL accounted for 59.13%. Primary PL accounted for 78.31%.

**Table 1:** Demographic Characteristics of Study Subjects in the pregnancy loss Cohort Study (*n* = 1955)

Variables	Number (n)		Variables	Number (n)
Age, years	30.51 ± 4.41		Live birth	
Age, years			No	1555 (79.54%)
< 25	106 (5.42%)		Yes	400 (20.46%)
25~	769 (39.34%)		Delivery method	
30~	753 (38.52%)		Vaginal delivery	268 (67.09%)
35~	327 (16.73%)		Caesarean section	132 (32.91%)
First pregnancy age	26.41 ± 3.74		Birth defects	
< 25	543 (28.30%)		No	1882 (96.27%)
25~	1046 (54.51%)		Yes	73 (3.73%)
30~	284 (14.80%)		Ectopic pregnancy	
35~	46 (2.40%)		No	1864 (95.35%)
BMI, kg/m^2^	22.39 ± 3.24		Yes	91 (4.65%)
BMI, kg/m^2^			Hydatidiform mole	
< 18.5	155 (8.07%)		No	1935 (98.98%)
18.5~	1264 (65.80%)		Yes	20 (1.02%)
24~	395 (20.56%)		Menarche age	13.54 ± 1.29
28~	107 (5.57%)		Menstrual cycle	
Total pregnancy numbers	2.36 ± 1.26		Regular	1636 (83.68%)
1	531 (27.16%)		Irregular	319 (16.32%)
2	701 (35.86%)		Dysmenorrhea	
3	414 (21.18%)		no	760 (38.87%)
4	184 (9.41%)		mild	885 (45.27%)
≥ 5	125 (6.39%)		moderate	216 (11.05%)
Pregnancy loss numbers	1.89 ± 1.00		severe	94 (4.81%)
1	799 (40.87%)		previous pelvic surgery	
2	750 (38.36%)		No	1693 (86.60%)
3	278 (14.22%)		Yes	262 (13.40%)
≥ 4	128 (6.55%)		Degree of education	
Induced abortion			Primary school degree	57 (2.92%)
0	1727 (88.34%)		High school degree	606 (31.00%)
1	179 (9.16%)		A college degree	1236 (63.22%)
≥ 2	49 (2.51%)		Graduate degree	56 (2.86%)
Pregnancy loss type			Ethnic	
Primary	1531 (78.31%)		Han	1763 (90.18%)
Secondary	424 (21.69%)		Hui	110 (5.63%)
			Zang	36 (1.84%)
			others	46 (2.35%)

Continuous variables are described as the mean ± standard deviation, categorical variables are expressed as numbers and percentages

The total number of cumulative pregnancies (defined as the total number of pregnancies at the time of the first visit for all patients, excluding the current already pregnant at the time of the first visit) was 4606, of which 3696 were PLs, 445 were live births, 251 were induced abortions, 101 were ectopic pregnancies, 20 were hydatidiform moles, 75 were birth defects, and 18 were others (Supplementary Fig. 1).

At the time of the first visit, 1,593 patients were currently non-pregnant, preparing for their next pregnancy and seeking help. There were also 362 patients who had also experienced at least one previous PL but sought treatment after their current pregnancy was confirmed, who were already pregnant at the time of the first visit.

### Reproductive status in different age, BMI, pregnancy loss numbers groups in the study

The survey showed that in different age groups (< 25, 25–29, 30–34, ≥ 35), the BMI, total pregnancy numbers, PL numbers and first pregnancy age were increased with age and the difference was statistically significant (*P* < 0.001). With the increase of age, the proportion of the types of secondary PL and the proportion of those who experienced induced abortion, live birth, cesarean section and pelvic surgery are increased (*P* < 0.001). The rate of ectopic pregnancies was higher in the 30–35 age group. With the increase of age, the proportion of women with regular periods increases, while the number of women with moderate or severe dysmenorrhea decreases (Supplementary Table 1). In different BMI groups (< 18.5 kg/m^2^, 18.5–23.9 kg/m^2^, 24.0-27.9 kg/m^2^, ≥ 28 kg/m^2^), there were differences in patients age and first pregnancy age. In addition, with the increase of BMI, the age of menarche was slightly earlier (*P* = 0.003). And the incidence of pelvic surgery was lowest in the normal-weight group (*P* < 0.001) (Supplementary Table 2). In different PL numbers groups (1, 2, 3, ≥ 4), the total pregnancy numbers and age were increased with the number of PL, the first pregnancy age was decreased with the number of PL (*P* < 0.001). With the increase of the number of PL, the proportion of secondary PL, live birth and regular menstruation are increased (*P* < 0.05) (Supplementary Table 3).

### The follow-up results of 1955 patients

**Fig. 1:**
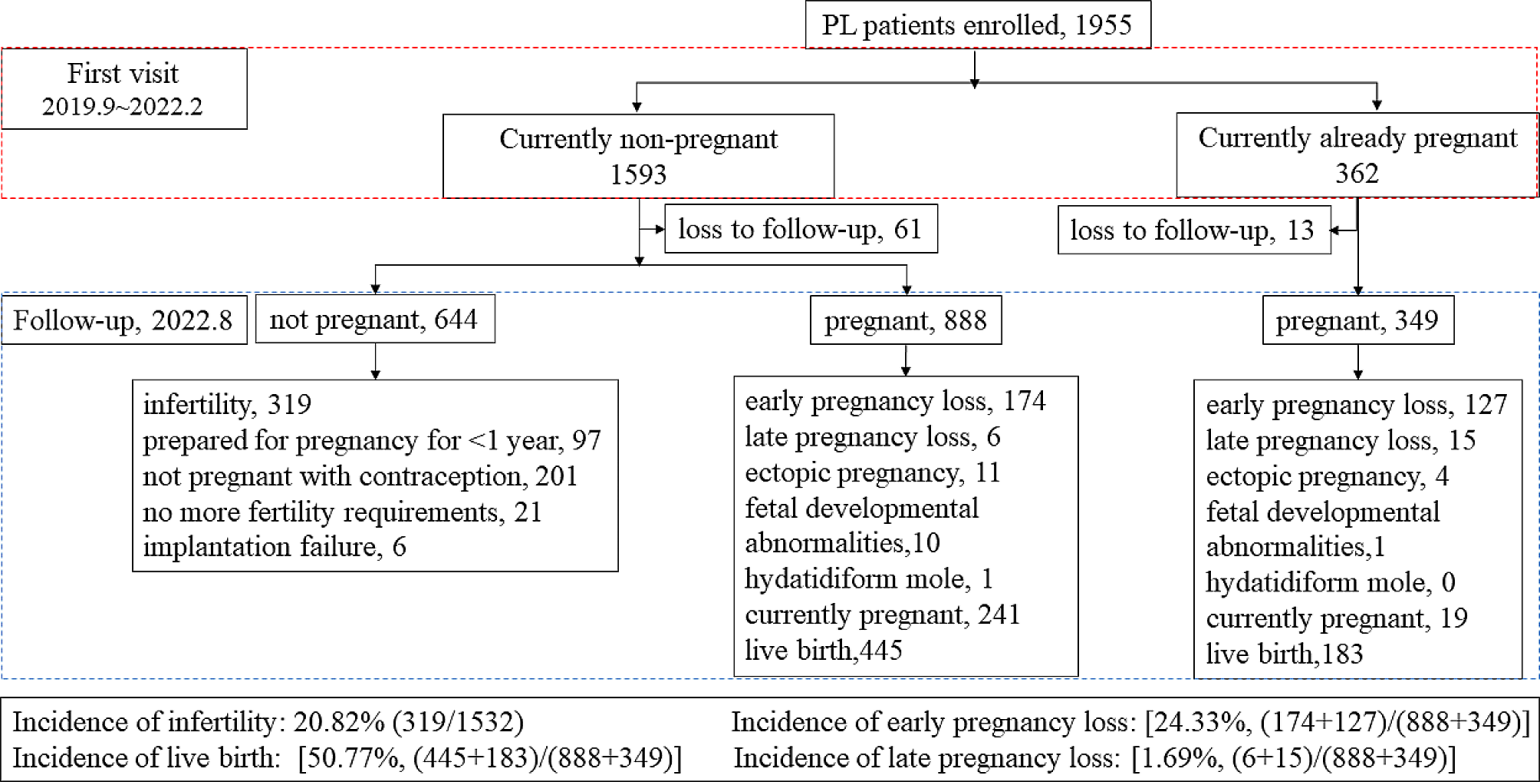
Flow diagram of the patients selected for the study

Figure 1. shows that during follow-up, 74 cases were refused to accept follow-up. Of the remaining 1881 patients, 1532 were non-pregnant at the time of consultation and 349 were already pregnant at the time of consultation. In a follow-up study of 1,532 non-pregnant women, we found that 644 patients who were not pregnant, of whom 319 patients had been diagnosed as infertile for more than 1 year without contraception. A total of 888 women experienced a second pregnancy, of which 174 had early PL, 6 had late PL, and 445 had a live birth. In the follow-up study of 349 pregnant women, we found that there were 127 women experienced their next early PL, 15 had late PL, and 183 had a live birth. Among all patients, the incidence of subsequent infertility was 20.82% (319/1532), the incidence of early PL was 24.33% [(174 + 127)/ (888 + 349)], and the incidence of late PL was 1.69% [(6 + 15)/ (888 + 349)]. The live birth rate was 50.77% [(445 + 183)/ (888 + 349)].

### Maternal and infant complications in patients with live birth in a subsequent pregnancy

**Fig. 2:**
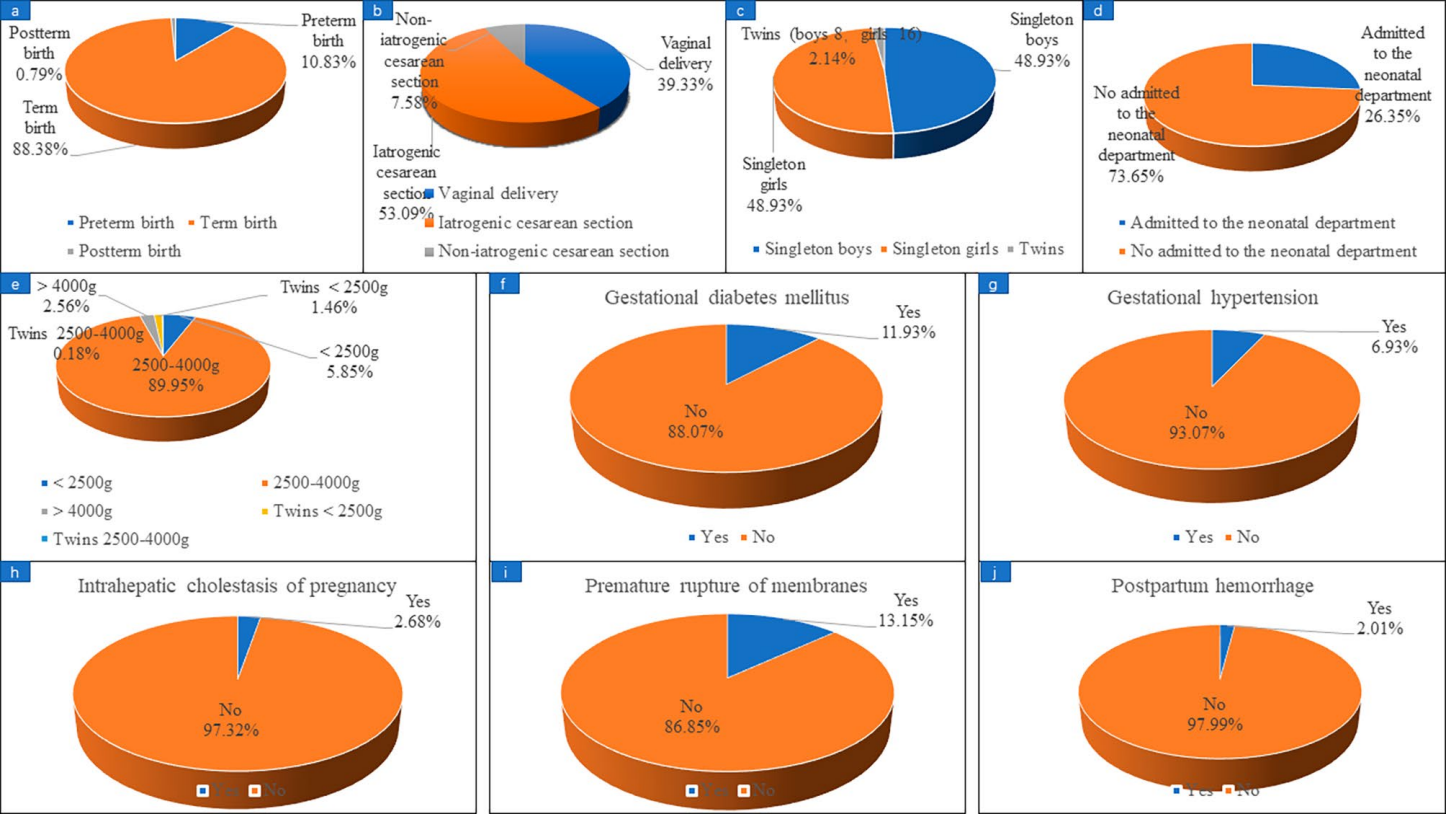
Maternal and infant complications in patients with live birth in subsequent pregnancy. **(a)** preterm birth; **(b)** delivery method; **(c)** gender of newborn; **(d)** newborns admitted to the neonatal department; **(e)** neonatal weight; **(f)** gestational diabetes mellitus; **(g)** gestational hypertension; **(h)** intrahepatic cholestasis of pregnancy; **(i)** premature rupture; **(j)** postpartum hemorrhage

Fig. 2. shows that, in the study, 628 confirmed live births were reported as of August 2022, of which preterm birth occurred in 68 patients, accounting for 10.83%. A total of 567 women reported their mode of delivery, including 223 (39.33%) vaginal delivery and 344 (60.67%) cesarean section. There were 43 cases of cesarean section due to patients’ request which called non-iatrogenic cesarean Sect. (43/567, 7.58%) and 301 cases of cesarean section due to medical reasons which called iatrogenic cesarean Sect. (301/567, 53.09%). The gender of the newborns was reported in 562 cases, including 275 singleton boys and 275 singleton girls. 461 cases reported whether they had gestational diabetes mellitus, of which 55 cases were diagnosed with gestational diabetes mellitus, accounting for 11.93%; 476 cases reported whether they had gestational hypertension, and 33 cases (6.93%) were diagnosed. 447 cases were reported whether they had intrahepatic cholestasis of pregnancy, and 12 cases (2.68%) were diagnosed. 479 cases reported whether they had premature rupture, and 63 cases were confirmed, accounting for 13.15%. 298 cases reported whether they had postpartum hemorrhage, and 6 cases were confirmed, accounting for 2.01%.

### Whether the previous pregnancy status affects the patient’s subsequent pregnancy?

During follow-up, 319 patients were diagnosed with infertility after their last pregnancy loss, and 1237 patients were able to achieve a successful pregnancy. There was a significant difference in age between the infertility group and the successful pregnancy group (31.02 ± 4.79 vs. 30.16 ± 4.13, *P* < 0.001). There were also statistical differences between the infertility and successful pregnancy groups in the type of PL, the previous live birth and the delivery method, the previous birth defects. The age of first pregnancy and BMI were different, but not statistically significant. There were no statistical differences in the total pregnancy numbers, the previous PL numbers, the history of induced abortion, ectopic pregnancy, hydatidiform mole, menarche age, menstrual cycle, dysmenorrhea or not, previous pelvic surgery, the last pregnancy termination method between the infertility group and the successful pregnancy group (Table 2). The *P* < 0.1 of the variables were included in the logistic regression and found that, increasing age (OR 1.08, 95% CI 1.04–1.13) and previous cesarean delivery history (OR 2.46, 95% CI 1.27–4.76) were risk factors for subsequent infertility in patients with PL (Table 3).

**Table 2:** Effect of previous pregnancy status on subsequent pregnancy outcomes

Variables	Successful Pregnancy Group *n* = 1237	Infertility group *n* = 319	P_1_	Live birth group *n* = 628	Non-live birth group *n* = 349	P_2_	Ongoing pregnancy group *n* = 756	Pregnancy loss group *n* = 322	P_3_
Age	30.16 ± 4.13	31.02 ± 4.79	< 0.001	29.86 ± 4.03	30.97 ± 4.49	< 0.001	29.89 ± 4.00	30.94 ± 4.54	< 0.001
First pregnancy age	26.55 ± 3.59	26.11 ± 4.09	0.060	26.30 ± 3.44	27.10 ± 3.89	0.001	26.31 ± 3.42	27.04 ± 3.92	0.002
BMI, kg/m^2^	22.29 ± 3.08	22.67 ± 3.82	0.065	22.19 ± 2.96	22.77 ± 3.31	0.005	22.17 ± 2.97	22.81 ± 3.30	0.002
Total pregnancy numbers	2.28 ± 1.22	2.32 ± 1.27	0.670	2.24 ± 1.17	2.39 ± 1.32	0.080	2.24 ± 1.17	2.35 ± 1.31	0.178
Previous pregnancy loss numbers	1.88 ± 0.97	1.80 ± 0.99	0.186	1.84 ± 0.95	2.00 ± 1.04	0.011	1.83 ± 0.96	1.97 ± 1.04	0.031
Pregnancy interval	-	-	-	17.02 ± 15.32	16.03 ± 12.27	0.318	17.06 ± 15.01	15.75 ± 11.77	0.177
Pre-pregnancy interventions	-	-	-			< 0.001			< 0.001
No	-	-	-	183 (55.45%)	147 (44.55%)		202 (58.72%)	142 (41.28%)	
Yes	-	-	-	445 (68.78%)	202 (31.22%)		554 (75.48%)	180 (24.52%)	
Pregnancy type			0.003			0.752			0.757
Primary	1005 (81.05%)	235 (18.95%)		504 (64.04%)	283 (35.96%)		609 (69.92%)	262 (30.08%)	
Secondary	232 (73.42%)	84 (26.58%)		124 (65.26%)	66 (34.74%)		147 (71.01%)	60 (28.99%)	
Induced abortion			0.883			0.882			0.858
No	1101 (79.55%)	283 (20.45%)		565 (64.35%)	313 (35.65%)		671 (70.04%)	287 (29.96%)	
Yes	136 (79.07%)	36 (20.93%)		63 (63.64%)	36 (36.36%)		85 (70.83%)	35 (29.17%)	
Previous live birth			0.003			0.784			0.774
No	1020 (80.95%)	240 (19.05%)		512 (64.08%)	287 (35.92%)		619 (69.94%)	266 (30.06%)	
Yes	217 (73.31%)	79 (26.69%)		116 (65.17%)	62 (34.83%)		137 (70.98%)	56 (29.02%)	
Previous delivery method			0.011			0.630			0.378
Vaginal delivery	137 (68.53%)	62 (31.47%)		72 (67.29%)	35 (32.71%)		89 (73.55%)	32 (26.45%)	
Caesarean section	80 (82.47%)	17 (17.53%)		44 (63.77%)	25 (36.23%)		48 (67.61%)	23 (32.39%)	
Previous birth defects			0.042			0.186			0.269
No	1200 (79.89%)	302 (20.11%)		606 (63.92%)	342 (36.08%)		730 (69.86%)	315 (30.14%)	
Yes	37 (68.52%)	17 (31.48%)		22 (75.86%)	7 (24.14%)		26 (78.79%)	7 (21.21%)	
Previous ectopic pregnancy			0.114			0.442			0.218
No	1182 (79.86%)	298 (20.14%)		598 (64.03%)	336 (35.97%)		720 (69.77%)	312 (30.23%)	
Yes	55 (72.37%)	21 (27.63%)		30 (69.77%)	13 (30.23%)		36 (78.26%)	10 (21.74%)	
Previous hydatidiform mole			0.347			0.692			0.819
No	1227 (79.42%)	318 (20.58%)		624 (64.33%)	346 (35.67%)		750 (70.16%)	319 (29.84%)	
Yes	10 (90.91%)	1 (9.09%)		4 (57.14%)	3 (42.86%)		6 (66.67%)	3 (33.33%)	
Menarche age	13.51 ± 1.25	13.57 ± 1.28	0.428	13.43 ± 1.21	13.56 ± 1.31	0.133	13.47 ± 1.22	13.58 ± 1.33	0.230
Menstrual cycle			0.658			0.397			0.475
Regular	1043 (79.68%)	266 (20.32%)		533 (64.84%)	289 (35.16%)		640 (70.56%)	267 (29.44%)	
Irregular	194 (78.54%)	53 (21.46%)		95 (61.29%)	60 (38.71%)		116 (67.84%)	55 (32.16%)	
Dysmenorrhea			0.689			0.083			0.120
no	482 (79.02%)	128 (20.98%)		241 (63.09%)	141 (36.91%)		284 (68.77%)	129 (31.23%)	
mild	551 (78.94%)	147 (21.06%)		297 (67.81%)	141 (32.19%)		362 (73.13%)	133 (26.87%)	
moderate	144 (82.76%)	30 (17.24%)		63 (55.26%)	51 (44.74%)		75 (62.50%)	45 (37.50%)	
severe	60 (81.08%)	14 (18.92%)		27 (62.79%)	16 (37.21%)		35 (70.00%)	15 (30.00%)	
Previous pelvic surgery			0.352			0.183			0.136
No	1086 (79.85%)	274 (20.15%)		541 (63.50%)	311 (36.50%)		659 (69.37%)	291 (30.63%)	
Yes	151 (77.04%)	45 (22.96%)		87 (69.60%)	38 (30.40%)		97 (75.78%)	31 (24.22%)	
Last pregnancy termination method			0.977			0.949			0.857
No intervention	380 (30.72%)	103 (32.29%)		198 (64.92%)	107 (35.08%)		233 (70.61%)	97 (29.39%)	
Medical abortion	125 (10.11%)	32 (10.03%)		61 (61.62%)	38 (38.38%)		73 (66.97%)	36 (33.03%)	
Surgical abortion	550 (44.46%)	138 (43.26%)		274 (63.72%)	156 (36.28%)		338 (69.55%)	148 (30.45%)	
Medical + surgical abortion	132 (10.67%)	32 (10.03%)		66 (66.67%)	33 (33.33%)		79 (73.15%)	29 (26.85%)	
Induced labor	50 (4.04%)	14 (4.39%)		29 (65.91%)	15 (34.09%)		33 (73.33%)	12 (26.67%)	

P1: Successful Pregnancy Group vs. Infertility group;P2: Live birth group vs. non-live birth group; P3: Ongoing pregnancy group vs. Pregnancy loss group

**Table 3:** Logistic regression of reproductive risk for infertility

Variables	OR	95%CI Low	95%CI Upp	***P***-value
Age	1.081	1.038	1.127	0.000
First pregnancy age	0.925	0.884	0.967	0.101
BMI, kg/m^2^	1.028	0.989	1.069	0.156
Pregnancy type (Secondary)	1.187	0.442	3.193	0.734
Live birth (Yes)	1.821	0.581	5.713	0.304
Delivery method (Caesarean section)	2.456	1.269	4.755	0.008
Birth defects (Yes)	0.552	0.277	1.100	0.091

### Whether the previous pregnancy status affects the live birth in subsequent pregnancy?

Of the 1237 women who had subsequent pregnancies, 977 had final pregnancy outcomes, including 628 live births and 349 non-live births. We found that the age, age at first pregnancy, BMI, and previous pregnancy loss numbers were lower in the live birth group than in the non-live birth group. Pre-pregnancy intervention increased live births compared to without pre-pregnancy intervention. Total pregnancy numbers were different but not statistically significant between the live birth group and the non-live birth group. There were no statistical differences in the total pregnancy numbers, the pregnancy interval, the pregnancy type, the history of induced abortion, ectopic pregnancy, hydatidiform mole, menarche age, menstrual cycle, dysmenorrhea or not, previous pelvic surgery, the last pregnancy termination method between the live birth group and the non-live birth group (Table 2). In logistic regression analysis, we found that age (OR 1.06, 95% CI 1.03–1.10), age at first pregnancy (OR 1.06, 95% CI 1.03–1.10), BMI (OR 1.06, 95% CI 1.02–1.11), previous pregnancy loss numbers (OR 1.18, 95% CI 1.04–1.57) and without pre-pregnancy intervention (OR 1.77, 95% CI 1.35–2.24) were risk factors for non-live birth (Table 4).

**Table 4:** Logistic regression of reproductive risk for non-live birth

Variables	OR	95%CI Low	95%CI Upp	***P***-value
Age	1.064	1.031	1.097	< 0.001
First pregnancy age	1.063	1.025	1.103	0.001
BMI, kg/m^2^	1.062	1.018	1.108	0.005
Total pregnancy numbers	1.098	0.988	1.221	0.081
Previous pregnancy loss numbers	1.183	1.038	1.573	0.012
Pre-pregnancy interventions (No)	1.770	1.346	2.236	< 0.001

### Whether the previous pregnancy status affects the pregnancy loss in subsequent pregnancy?

Of the 1237 women who had subsequent pregnancies, 322 had confirmed subsequent pregnancy losses and 756 had pregnancies that were > 24 W, which was considered an ongoing pregnancy. We found that age, age at first pregnancy, BMI, and previous pregnancy loss numbers were higher in the pregnancy loss group than in the ongoing pregnancy group. Pre-pregnancy intervention decreased pregnancy loss compared to without pre-pregnancy intervention. There were no statistical differences in the total pregnancy numbers, the pregnancy interval, the pregnancy type, the history of induced abortion, ectopic pregnancy, hydatidiform mole, menarche age, menstrual cycle, dysmenorrhea or not, previous pelvic surgery, the last pregnancy termination method between the pregnancy loss group and the ongoing pregnancy group (Table 2). In logistic regression analysis, we found that age (OR 1.06, 95% CI 1.03–1.09), age at first pregnancy (OR 1.06, 95% CI 1.02–1.09), BMI (OR 1.07, 95% CI 1.02–1.11), previous pregnancy loss numbers (OR 1.15, 95% CI 1.02–1.31) and without pre-pregnancy intervention (OR 2.16, 95% CI 1.65–2.84) were risk factors for PL (Table 5).

**Table 5:** Logistic regression of reproductive risk for subsequence pregnancy loss

Variables	OR	95%CI Low	95%CI Upp	***P***-value
Age	1.061	1.029	1.094	< 0.001
First pregnancy age	1.058	1.020	1.098	0.003
BMI, kg/m^2^	1.068	1.024	1.114	0.002
Total pregnancy numbers	1.075	0.967	1.195	0.178
Previous pregnancy loss numbers	1.151	1.012	1.309	0.032
Pre-pregnancy interventions (No)	2.164	1.647	2.842	< 0.001

## Discussion

The incidence of PL has been increasing in recent years, but few studies have summarized the reproductive status of patients with previous PL. Our study summarized the distribution of pregnancies in 1955 pregnancy loss patients and followed them for subsequent pregnancy outcomes. We found that patients with PL also had other adverse pregnancy events, such as birth defects (3.73%), ectopic pregnancy (4.65%) and hydatidiform mole (1.02%). But none of this have an effect on subsequent pregnancies in our analysis. Of the 1955 women with PL, 20.46% had a previous live birth, of which 32.91% were delivered by cesarean section, which increased the risk of subsequent infertility in women with PL, but had no effect on the ongoing pregnancy and live birth in subsequent pregnancies. In recent years, the relationship between cesarean scar uterus and subsequent secondary infertility has been gradually recognized, but the specific mechanism is not clear [20, 21]. Nobuta et al. found that a cause of secondary infertility in women with cesarean scar syndrome may be chronic inflammation of the uterine cavity [22]. We also found that prior induced abortion, mode of termination of the last pregnancy, age at menarche, menstrual cycle, and level of dysmenorrhea had no effect on subsequent pregnancy outcomes. However, previous studies have found that the risk of spontaneous abortion decreases with the increase in the number of induced abortions among female workers in the Jinchang Cohort [7]. This is not consistent with our results. The possible reason is that the reference population was derived from all female workers in the Jinchang cohort in China, most of whom had normal reproductive function. In contrast, all the patients in our study were women of childbearing age who had experienced at least one pregnancy loss.

Our study found that age is an important risk factor in the occurrence of infertility after PL, also resulting in an increased risk of pregnancy loss and a decreased live birth in subsequent pregnancies. The association between female age and RPL has been consistently demonstrated in several studies. The age-related risk of pregnancy loss followed a J-shaped curve, with the lowest risk at ages 25 to 29 years, an increase in risk among women 30 to 35 years of age, and then a sharp rise in risk among women 40 to 44 years of age [8].

Age at first pregnancy, BMI, and the number of previous PL were also key indicators of subsequent pregnancy failure. Based on a computer-simulated fertility model, couples should start trying to conceive when the woman is 31 or less to have at least a 90% chance of having a two-child family, and if IVF is not feasible, couples should start planning no later than 27. In order to achieve a one-child family, couples should start trying before the age of 32, or 35 if IVF is an option [23].

Our study found that approximately 26.13% (140/658) of prior PL patients were overweight/obesity, which is higher than the pre-pregnancy overweight/obesity rates found in a birth cohort in Shanghai (19.06% (106/556)) [24]. But in the USA, a 2009–2010 survey indicated that 55.8% of women of childbearing age were overweight or obese, defined as having a BMI of 25 or higher, significantly higher than our research found [25]. There are also variations in the threshold of BMI for pregnancy. Zhang et al. reported that, a BMI of 24.0 kg/m^2^ or greater was associated with an increased risk of RPL, but Lo and colleagues demonstrated that maternal obesity (BMI ≥ 30.0 kg/m^2^) significantly increased the risk of miscarriage in couples with unexplained RPL and there was no increased risk in women with overweight and underweight [10, 26]. This suggests that BMI reference ranges should be tailored to patient geographic region and disease status.

The impact of the number of previous PL on the chance of live birth has been investigated in several cohort studies. The risk of PL during a second pregnancy is associated with the number of PL. The risk is about 20% after one PL, 28% after two PLs, and 43% after three or more PLs [27, 28]. In a nested cohort, it was demonstrated that the number of prior miscarriages was a determinant both for time to live birth and cumulative incidence of live birth [29, 30]. It is worth noting that for secondary URPL, only consecutive PL after the birth influenced the subsequent prognosis, while the number of losses prior to the birth did not affect the prognosis in the next pregnancy [31].

Finally, we found that individualized pre-pregnancy intervention increased the rate of live birth and decreased the rate of PL in subsequent pregnancies. These individualized pre-pregnancy interventions were based on patient clinical examination findings, including treatment for endocrine abnormalities, prethrombotic state, immune disorders, antiphospholipid antibody syndrome, and lifestyle modification before subsequence pregnancy. Study found that a combination of heparin and aspirin treatment can improve the APS and recurrent pregnancy loss of the pregnancy outcomes of women but add corticosteroids (e.g., prednisone), cannot improve live birth rates, and increase the risk of obstetric diseases, such as premature delivery, preeclampsia, gestational diabetes, enter the neonatal intensive care unit [32, 33]. Patients with RPL who have overt hypothyroidism before or during the first trimester should be treated with levothyroxine (thyroid hormone replacement therapy). However, levothyroxine did not improve pregnancy outcomes in patients with subclinical hypothyroidism [34]. For immune diseases, the treatment of intravenous immune globulin (IVIG) is still controversial [35, 36]. At present, there are still some controversies and uncertainties in the treatment of PL patients, and further standardized treatment is needed. In addition, RPL is an independent risk factor for women’s long-term increased incidence of malignant tumors (such as breast cancer and cervical cancer) and cardiovascular diseases [37]. Therefore, we should give individualized pre-pregnancy intervention to patients with PL not only to improve the subsequent pregnancy outcome, but also to potentially reduce the risk of long-term complications.

Our study still has some limitations. We did not capture complications for all patients who had live births. Due to the individualization of pre-pregnancy treatment, the diagnosis and treatment process were not recorded in detail. However, we are in the process of establishing pregnancy-loss specific cohorts, and the management of future patients will be more careful.

## Conclusion

Maternal age and a history of cesarean section in a previous pregnancy are key factors for subsequent failure to achieve a successful pregnancy in patients with PL. Maternal age, age at first pregnancy, BMI, number of previous PL and pre-pregnancy treatment are the key factors affecting subsequent PL.

### Electronic supplementary material

Below is the link to the electronic supplementary material.

Supplementary Material 1

## Data Availability

The data that support the findings of this study are available from the corresponding author upon reasonable request.
