# Supplementary material for: Reproductive factors and subsequent pregnancy outcomes in patients with prior pregnancy loss
Source: BMC Pregnancy Childbirth. 2024 Mar 25;24:219. doi: 10.1186/s12884-024-06422-1 (PMC10964557; doi:10.1186/s12884-024-06422-1)
Supplement: Supplementary file 1 — Supplementary Material 1 [file 12884_2024_6422_MOESM1_ESM.docx]

**Supplementary figure 1.**

Distribution of cumulative reproductive status in 1955 women with pregnancy loss.

**Supplementary table 1.**

Reproductive Status in Different Age Groups in the Study (n = 1955).

|  | <25 | 25~ | 30~ | 35~ | P |
| --- | --- | --- | --- | --- | --- |
| Numbers | n =106 | n =769 | n =753 | n =327 |  |
| Total pregnancy numbers | 1.92 ± 0.92 | 2.02 ± 1.06 | 2.43 ± 1.23 | 3.11 ± 1.46 | <0.001 |
| Pregnancy loss numbers | 1.64 ± 0.80 | 1.76 ± 0.90 | 1.95 ± 1.03 | 2.13 ± 1.16 | <0.001 |
| First pregnancy age | 20.49 ± 2.02 | 25.10 ± 2.40 | 27.47 ± 2.98 | 29.03 ± 4.90 | <0.001 |
| BMI, kg/m^2^ | 22.05 ± 4.05 | 22.06 ± 3.20 | 22.45 ± 3.18 | 23.15 ± 3.08 | <0.001 |
| Pregnancy type |  |  |  |  | <0.001 |
| Primary | 98 (92.45%) | 699 (90.90%) | 574 (76.23%) | 160 (48.93%) |  |
| Secondary | 8 (7.55%) | 70 (9.10%) | 179 (23.77%) | 167 (51.07%) |  |
| Induced abortion |  |  |  |  | <0.001 |
| No | 95 (89.62%) | 697 (90.64%) | 679 (90.17%) | 256 (78.29%) |  |
| Yes | 11 (10.38%) | 72 (9.36%) | 74 (9.83%) | 71 (21.71%) |  |
| Live birth |  |  |  |  | <0.001 |
| No | 100 (94.34%) | 712 (92.59%) | 584 (77.56%) | 159 (48.62%) |  |
| Yes | 6 (5.66%) | 57 (7.41%) | 169 (22.44%) | 168 (51.38%) |  |
| Delivery method |  |  |  |  | <0.001 |
| Vaginal delivery | 100 (94.34%) | 712 (92.59%) | 584 (77.56%) | 159 (48.62%) |  |
| Caesarean section | 6 (5.66%) | 57 (7.41%) | 169 (22.44%) | 168 (51.38%) |  |
| Birth defects |  |  |  |  | 0.940 |
| No | 103 (97.17%) | 739 (96.10%) | 726 (96.41%) | 314 (96.02%) |  |
| Yes | 3 (2.83%) | 30 (3.90%) | 27 (3.59%) | 13 (3.98%) |  |
| Ectopic pregnancy |  |  |  |  | 0.021 |
| No | 101 (95.28%) | 747 (97.14%) | 707 (93.89%) | 309 (94.50%) |  |
| Yes | 5 (4.72%) | 22 (2.86%) | 46 (6.11%) | 18 (5.50%) |  |
| Hydatidiform mole |  |  |  |  | 0.481 |
| No | 104 (98.11%) | 763 (99.22%) | 743 (98.67%) | 325 (99.39%) |  |
| Yes | 2 (1.89%) | 6 (0.78%) | 10 (1.33%) | 2 (0.61%) |  |
| Menarche age | 13.54 ± 1.34 | 13.59 ± 1.28 | 13.46 ± 1.25 | 13.62 ± 1.36 | 0.156 |
| Menstrual cycle |  |  |  |  | <0.001 |
| Regular | 82 (77.36%) | 621 (80.75%) | 637 (84.59%) | 296 (90.52%) |  |
| Irregular | 24 (22.64%) | 148 (19.25%) | 116 (15.41%) | 31 (9.48%) |  |
| Dysmenorrhea |  |  |  |  | <0.001 |
| no | 36 (33.96%) | 275 (35.76%) | 290 (38.51%) | 159 (48.62%) |  |
| mild | 40 (37.74%) | 356 (46.29%) | 349 (46.35%) | 140 (42.81%) |  |
| moderate | 19 (17.92%) | 102 (13.26%) | 77 (10.23%) | 18 (5.50%) |  |
| severe | 11 (10.38%) | 36 (4.68%) | 37 (4.91%) | 10 (3.06%) |  |
| Previous pelvic surgery |  |  |  |  | <0.001 |
| No | 95 (89.62%) | 685 (89.08%) | 652 (86.59%) | 261 (79.82%) |  |
| Yes | 11 (10.38%) | 84 (10.92%) | 101 (13.41%) | 66 (20.18%) |  |

**Supplementary table 2.**

Reproductive Status in Different BMI Groups in the Study (n = 1921).

|  | <18.5 | 18.5~ | 24~ | 28~ | P |
| --- | --- | --- | --- | --- | --- |
| Numbers | 155 | 1264 | 395 | 107 |  |
| Total pregnancy numbers | 2.19 ± 1.01 | 2.36 ± 1.24 | 2.41 ± 1.38 | 2.35 ± 1.33 | 0.318 |
| Pregnancy loss numbers | 1.81 ± 0.81 | 1.91 ± 1.01 | 1.88 ± 1.08 | 1.85 ± 0.93 | 0.654 |
| Age | 29.19 ± 4.41 | 30.30 ± 4.20 | 31.63 ± 4.86 | 30.75 ± 4.30 | <0.001 |
| First pregnancy age | 25.76 ± 3.82 | 26.35 ± 3.61 | 26.93 ± 4.02 | 25.98 ± 3.97 | 0.003 |
| Pregnancy type |  |  |  |  | 0.794 |
| Primary | 125 (80.65%) | 994 (78.64%) | 305 (77.22%) | 82 (76.64%) |  |
| Secondary | 30 (19.35%) | 270 (21.36%) | 90 (22.78%) | 25 (23.36%) |  |
| Induced abortion |  |  |  |  | 0.738 |
| No | 138 (89.03%) | 1123 (88.84%) | 343 (86.84%) | 95 (88.79%) |  |
| Yes | 17 (10.97%) | 141 (11.16%) | 52 (13.16%) | 12 (11.21%) |  |
| Live birth |  |  |  |  | 0.608 |
| No | 128 (82.58%) | 1010 (79.91%) | 308 (77.97%) | 83 (77.57%) |  |
| Yes | 27 (17.42%) | 254 (20.09%) | 87 (22.03%) | 24 (22.43%) |  |
| Delivery method |  |  |  |  | 0.235 |
| Vaginal delivery | 18 (66.67%) | 175 (68.90%) | 58 (67.44%) | 11 (47.83%) |  |
| Caesarean section | 9 (33.33%) | 79 (31.10%) | 28 (32.56%) | 12 (52.17%) |  |
| Birth defects |  |  |  |  | 0.783 |
| No | 150 (96.77%) | 1214 (96.04%) | 383 (96.96%) | 102 (95.33%) |  |
| Yes | 5 (3.23%) | 50 (3.96%) | 12 (3.04%) | 5 (4.67%) |  |
| Ectopic pregnancy |  |  |  |  | 0.106 |
| No | 150 (96.77%) | 1213 (95.97%) | 368 (93.16%) | 102 (95.33%) |  |
| Yes | 5 (3.23%) | 51 (4.03%) | 27 (6.84%) | 5 (4.67%) |  |
| Hydatidiform mole |  |  |  |  | 0.991 |
| No | 153 (98.71%) | 1251 (98.97%) | 391 (98.99%) | 106 (99.07%) |  |
| Yes | 2 (1.29%) | 13 (1.03%) | 4 (1.01%) | 1 (0.93%) |  |
| Menarche age | 13.66 ± 1.33 | 13.59 ± 1.27 | 13.41 ± 1.28 | 13.21 ± 1.35 | 0.003 |
| Menstrual cycle |  |  |  |  | 0.160 |
| Regular | 129 (83.23%) | 1069 (84.57%) | 325 (82.28%) | 82 (76.64%) |  |
| Irregular | 26 (16.77%) | 195 (15.43%) | 70 (17.72%) | 25 (23.36%) |  |
| Dysmenorrhea |  |  |  |  | 0.557 |
| no | 57 (36.77%) | 475 (37.58%) | 171 (43.29%) | 41 (38.32%) |  |
| mild | 72 (46.45%) | 582 (46.04%) | 167 (42.28%) | 52 (48.60%) |  |
| moderate | 16 (10.32%) | 149 (11.79%) | 39 (9.87%) | 8 (7.48%) |  |
| severe | 10 (6.45%) | 58 (4.59%) | 18 (4.56%) | 6 (5.61%) |  |
| Previous pelvic surgery |  |  |  |  | <0.001 |
| No | 133 (85.81%) | 1124 (88.92%) | 317 (80.25%) | 88 (82.24%) |  |
| Yes | 22 (14.19%) | 140 (11.08%) | 78 (19.75%) | 19 (17.76%) |  |

(BMI data were missing for 34 patients)

**Supplementary table 3.**

Reproductive Status in Different pregnancy loss numbers Groups in the Study (n = 1955).

|  | 1 | | 2 | | 3 | | ≥4 | P |
| --- | --- | --- | --- | --- | --- | --- | --- | --- |
| Numbers | 799 | 750 | | 278 | | 128 | |  |
| Total pregnancy numbers | 1.48 ± 0.81 | 2.43 ± 0.76 | | 3.45 ± 0.77 | | 4.98 ± 1.03 | | <0.001 |
| Age | 29.99 ± 4.33 | 30.44 ± 4.23 | | 31.47 ± 4.61 | | 32.12 ± 4.83 | | <0.001 |
| First pregnancy age | 26.64 ± 3.82 | 26.43 ± 3.51 | | 26.42 ± 3.93 | | 24.87 ± 3.83 | | <0.001 |
| BMI | 22.38 ± 3.29 | 22.25 ± 3.20 | | 22.62 ± 3.26 | | 22.72 ± 3.15 | | 0.244 |
| Pregnancy type |  |  | |  | |  | | 0.005 |
| Primary | 651 (81.48%) | 583 (77.73%) | | 208 (74.82%) | | 89 (69.53%) | |  |
| Secondary | 148 (18.52%) | 167 (22.27%) | | 70 (25.18%) | | 39 (30.47%) | |  |
| Induced abortion |  |  | |  | |  | | 0.309 |
| No | 693 (86.73%) | 669 (89.20%) | | 249 (89.57%) | | 116 (90.62%) | |  |
| Yes | 106 (13.27%) | 81 (10.80%) | | 29 (10.43%) | | 12 (9.38%) | |  |
| Live birth |  |  | |  | |  | | 0.003 |
| No | 662 (82.85%) | 593 (79.07%) | | 208 (74.82%) | | 92 (71.88%) | |  |
| Yes | 137 (17.15%) | 157 (20.93%) | | 70 (25.18%) | | 36 (28.12%) | |  |
| Delivery method |  |  | |  | |  | | 0.320 |
| Vaginal delivery | 96 (70.07%) | 96 (61.94%) | | 48 (68.57%) | | 27 (75.00%) | |  |
| Caesarean section | 41 (29.93%) | 59 (38.06%) | | 22 (31.43%) | | 9 (25.00%) | |  |
| Birth defects |  |  | |  | |  | | 0.006 |
| No | 755 (94.49%) | 730 (97.33%) | | 273 (98.20%) | | 124 (96.88%) | |  |
| Yes | 44 (5.51%) | 20 (2.67%) | | 5 (1.80%) | | 4 (3.12%) | |  |
| Ectopic pregnancy |  |  | |  | |  | | 0.115 |
| No | 752 (94.12%) | 721 (96.13%) | | 270 (97.12%) | | 121 (94.53%) | |  |
| Yes | 47 (5.88%) | 29 (3.87%) | | 8 (2.88%) | | 7 (5.47%) | |  |
| Hydatidiform mole |  |  | |  | |  | | 0.282 |
| No | 789 (98.75%) | 745 (99.33%) | | 276 (99.28%) | | 125 (97.66%) | |  |
| Yes | 10 (1.25%) | 5 (0.67%) | | 2 (0.72%) | | 3 (2.34%) | |  |
| Menarche age | 13.54 ± 1.29 | 13.52 ± 1.26 | | 13.54 ± 1.31 | | 13.66 ± 1.36 | | 0.780 |
| Menstrual cycle |  |  | |  | |  | | <0.001 |
| Regular | 631 (78.97%) | 647 (86.27%) | | 243 (87.41%) | | 115 (89.84%) | |  |
| Irregular | 168 (21.03%) | 103 (13.73%) | | 35 (12.59%) | | 13 (10.16%) | |  |
| Dysmenorrhea |  |  | |  | |  | | 0.004 |
| no | 276 (34.54%) | 321 (42.80%) | | 113 (40.65%) | | 50 (39.06%) | |  |
| mild | 366 (45.81%) | 336 (44.80%) | | 124 (44.60%) | | 59 (46.09%) | |  |
| moderate | 108 (13.52%) | 61 (8.13%) | | 34 (12.23%) | | 13 (10.16%) | |  |
| severe | 49 (6.13%) | 32 (4.27%) | | 7 (2.52%) | | 6 (4.69%) | |  |
| Previous pelvic surgery |  |  | |  | |  | | 0.090 |
| No | 688 (86.11%) | 663 (88.40%) | | 239 (85.97%) | | 103 (80.47%) | |  |
| Yes | 111 (13.89%) | 87 (11.60%) | | 39 (14.03%) | | 25 (19.53%) | |  |
